# Improved Analgesic Effect of Paravertebral Blocks before and after Video-Assisted Thoracic Surgery: A Prospective, Double-Blinded, Randomized Controlled Trial

**DOI:** 10.1155/2019/9158653

**Published:** 2019-11-18

**Authors:** Lihua Chu, Xiaolin Zhang, Yaping Lu, Guohao Xie, Shengwen Song, Xiangming Fang, Baoli Cheng

**Affiliations:** ^1^Department of Anesthesiology, The First Affiliated Hospital, School of Medicine, Zhejiang University, Qingchun Road 79, Hangzhou 31003, China; ^2^Department of Anesthesiology, Hangzhou Red Cross Hospital, East Road 208, Hangzhou 31003, China; ^3^Department of Anesthesiology, The Jiaxing First Hospital, South Zhonghuan Road 1882, Jiaxing 314000, China

## Abstract

Despite being less invasive, patients who underwent video-assisted thoracic surgery (VATS) suffered considerable postoperative pain. Paravertebral block (PVB) was proven to provide effective analgesia in patients with VATS; however, there is no difference in pain relief between preoperative PVB and postoperative PVB. This study was aimed to investigate the analgesic efficacy of combination of preoperative and postoperative PVB on the same patient undergoing VATS. In this prospective, double-blinded, randomized controlled trial, 44 patients undergoing VATS were enrolled, and they received patient-controlled intravenous analgesia (PCIA) with sufentanil plus preoperative PVB (Group A, *n* = 15) or postoperative PVB (Group B, *n* = 15), or combination of preoperative and postoperative PVB (Group C, *n* = 14). The primary outcome was sufentanil consumption and PCIA press times in the first 24 hours postoperatively. Also, data of postoperative use of PCIA and visual analogue scale (VAS) were collected. In the first 24 hours postoperatively, median sufentanil consumption in Group C was 0 (0–34.75) *μ*g, which was much less than that in Group A (45.00 (33.00–47.00) *μ*g, *p*=0.005) and Group B (36 (20.00–50.00) *μ*g, *p*=0.023). Patients in Group C pressed less times of PCIA (0 (0–0) times) than patients in Group A (2 (1–6) times, *p* < 0.001) and Group B (2 (1–3) times, *p*=0.009). Kaplan–Meier analysis showed patients with combination of preoperative and postoperative PVB had a higher PCIA-free rate than patients with either technique alone (*p*=0.003). The VAS among the three groups was comparable postoperatively. The combination of both preoperative and postoperative PVB provides better analgesic efficacy during the early postoperative period and may be an alternative option for pain control after VATS. This trial is registered with ChiCTR1800017102.

## 1. Introduction

The video-assisted thoracic surgical (VATS) approach is a well-established method for lobectomy [1, 2]. Though it is considered less traumatic than thoracotomy, the management of postoperative pain remains a concern [3, 4]. The sources of the acute postoperative pain vary, including the surgical incision and chest drain insertion, as well as inflammation of adjacent chest wall structures [5]. Inadequate pain control in the early postoperative period can cause hypoxia, atelectasis, and pulmonary infections [6] and may lead to chronic neuralgia [7, 8]. Therefore, appropriate pain management is essential to improve outcomes of these patients.

There are several measures for pain control after VATS. Systemic opioids are used commonly but may result in oversedation and hypotension [9]. Intercostal nerve block can also provide analgesia, while its effectiveness seemed not superior to intraoperative incision site injection [10]. Epidural analgesia is effective in relieving pain [4]; however, it has risks of dural perforation, nerve lesions, epidural hematoma, and hypotension [11].

The effectiveness of PVB has been shown to be equal to that of epidural analgesia for postoperative pain [11] and has a lower incidence of side effects [10]. PVB has been easy to perform for the development of ultrasound-guided technique and proven to be effective analgesia in VATS, breast surgery, and cholecystectomy [12–15]. However, very few publications have evaluated the analgesia effect of the combination of preoperative and postoperative PVB on the same patient undergoing VATS. This prospective, double-blinded, randomized controlled study, aimed to compare the analgesic efficacy of preoperative or postoperative PVB versus the combination of both in patients undergoing VATS.

## 2. Patients and Methods

This study was a prospective, double-blinded, randomized controlled trial comparing analgesia effect in patients receiving preoperative or postoperative or a combination of them after VATS. The study protocol was approved by the Ethics Committee of the First Affiliated Hospital, School of Medicine, Zhejiang University (reference number 2017(17)), and written informed consents were obtained from all enrolled patients. This study was conducted at the First Affiliated Hospital, Zhejiang University, from March 1^st^, 2017, to May 31^st^, 2018. Patients in Group A received preoperative PVB and postoperative paravertebral injection (PVI) of normal saline plus patient-controlled intravenous analgesia (PCIA) with sufentanil. Patients in Group B received preoperative PVI of normal saline and postoperative PVB plus PCIA with sufentanil. Patients in Group C received a combination of both preoperative and postoperative PVB, as well as PCIA with sufentanil.

The sample size required was calculated choosing a difference of 24 *μ*g in sufentanil consumption as the minimum desired difference among the groups. Setting *α* = 0.05, assuming a standard deviation of 23 *μ*g (calculated in the preliminary experiment), and investigating 12 subjects per group, one can detect a significant difference of 24 *μ*g with a power of 0.8 (two-sided hypothesis).

Patients scheduled for elective VATS were screened for the present study. The inclusion criteria were as follows: aged from 18 to 80 years, American Society of Anesthesiologists' physical class of I–III and only one chest drain catheter insertion. Exclusion criteria were as follows: preoperative thoracic pain, continuous use of analgesics before surgery, radiotherapy on the chest before surgery, inability to express pain scores because of comorbidities such as mental retardation, allergy to ropivacaine, history of thoracic surgery, and surgical procedure including parietal pleura resection, pregnancy, or lactation.

### 2.1. Randomization and Blinding

Patients were randomized using a computer-generated randomization sequence, which was performed by an investigator who not involved in patient care. A nurse not involved in the study received sealed opaque envelopes that contained the allocation results and prepared 10 ml of 0.2% ropivacaine or normal saline in identical 10 mL syringes. Then, the syringe was given to the single anesthesiologist with 5 years' experience in pain management who conducted the PVB procedure. Patients' follow-up and data collection were conducted by another investigator who was also blinded to the group allocation.

### 2.2. Intraoperative Management

There was no premedication administrated. After arrival, the visual analogue scale (VAS) was explained to the patient. Then, electrocardiogram, invasive blood pressure, and oxygen saturation were monitored. When the total volume of administrated crystalloid reached to 4 mL/kg intravenously, anesthesia was induced with propofol 1.5–2 mg/kg and fentanyl 2.5 *μ*g/kg. After the patient lost consciousness, cisatracurium 0.15 mg/kg was administrated, and intubation was performed using a double-lumen tube. Intravenous propofol and remifentanil were used to maintain the general anesthesia and was adjusted to remain the bispectral index of 40–60. When both lungs were ventilated, the tidal volume was 6–8 mL/kg, and the ventilation frequency was adjusted to maintain an end-tidal carbon dioxide tension of 35–40 mmHg. During one-lung ventilation, the tidal volume was 6 mL/kg, and the ventilating frequency was adjusted to maintain an end-tidal carbon dioxide tension of 37–45 mmHg.

According to the site of lesion, the surgery was performed under two to three ports. A chest drain was inserted into the main port. After completion of the operation, pyridostigmine 1 mg and tropisetron 5 mg were injected to reverse the muscle relaxation and prevent postoperative nausea and vomiting (PONV). The patient was extubated after verifying a sufficient recovery of consciousness and spontaneous respiration. Analgesics were administered through the PCIA device (Hospira Inc, Lake Forest, IL, USA) when the VAS of the patient was no less than four (we defined it as PCIA start). A total of 100 *μ*g sufentanil and 15 mg tropisetron were mixed with 100 ml normal saline in each PCIA device. Continuous infusion was set to 2 ml/h (sufentanil 2 *μ*g/h), with a 1 ml bolus dose (sufentanil 1 *μ*g) and a 15 min lockout time. If PCIA alone could not provide sufficient pain control, 50 mg of flurbiprofen would be injected intravenously once for rescue analgesia.

### 2.3. Paravertebral Block

The PVI procedures (using 0.2% ropivacaine or normal saline) were performed at two time points which were before surgery (after induction of anesthesia) and after surgery (before extubation) for every patient. After positioning the patient in the lateral position and aseptically preparing the area required for the block, a 13 MHz high-frequency linear transducer (SonoSite Inc, Bothell, WA, USA) in a sterile sleeve was placed longitudinally at the midline of the T3 level. Then, the probe was moved laterally until the transverse process was detected, and the thoracic paravertebral space could be recognized. After confirming pleural movement, an 18G needle (Tuohy 18G; Braun, Melsungen, Germany) was inserted in a lateral-to-middle direction until the needle entered into the paravertebral space. After negative aspiration, 10 ml of 0.2% ropivacaine or normal saline was injected. The displacement of the parietal pleural by the liquid could be seen. After completion of the PVI, the M-mode ultrasound was used to observe whether there exists pneumothorax. The same procedure was applied at the T5 level.

### 2.4. Data Collection

The primary outcome was the sufentanil consumption infused by PCIA and the PCIA press times in the first 24 hours postoperatively. The use of PCIA during 72 hours after surgery was also collected.

The second outcome was the VAS during rest, during rotation of the homolateral arm, and while coughing at the time points of 1st, 8th, 24th, 48th, and 72nd postoperative hours. The number of patients that intravenously injected flurbiprofen for rescue analgesia was also collected. Adverse effects of hypotension (defined as the invasive blood pressure was beyond 80%–120% of preoperative values), arrhythmia, PONV, pleural puncture, dizziness, pruritus, and pneumonia were recorded.

### 2.5. Statistical Analysis

Statistical analysis was performed using SPSS version 20.0 (IBM Corp., Armonk, NY). Numerical data were presented as numbers (percentage) and analyzed using Fischer's exact test. Variables with a normal distribution were presented as mean ± standard deviation (SD) and compared using analysis of variance with LSD correction for post hoc multiple comparisons. Variables with a skewed distribution were presented as median (quartiles) and were compared using the Kruskall–Wallis H test. If results were statistically significant among the three groups, Nemeny test was further used for multiple two-group comparisons.

When analyzing the use of PCIA during the 72 hours postoperatively, the PCIA-free rate was assessed according to the Kaplan–Meier life-table analysis. Group A and Group B were merged into one group (Group A + B: patients with either preoperative or postoperative PVB). A log-rank test was used to compare the survival rates between patients with combination of preoperative and postoperative PVB (Group C) and patients with either of them (Group A + B).

Differences in measured results were considered significant if the *p* value was <0.05.

## 3. Results

**Figure 1:**
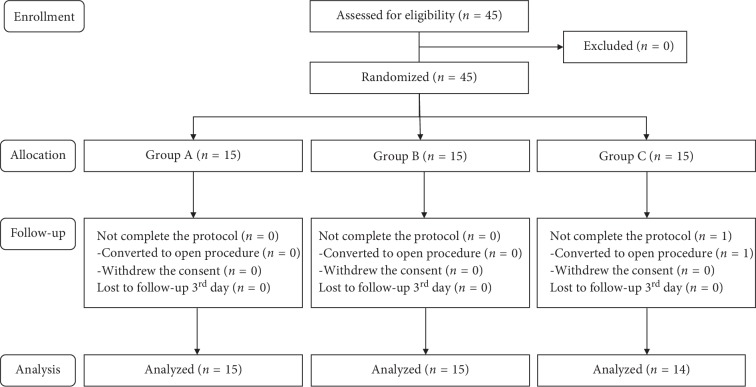
Patient flow diagram.

From March 1^st^, 2017, to May 31^st^, 2018, 45 patients assessed for eligibility were enrolled. One patient in Group C was excluded from analysis because of surgery conversion to open thoracotomy (Figure 1). Demographic and surgical data are listed in Table 1. Among the groups, there were no significant differences.

**Figure 2:**
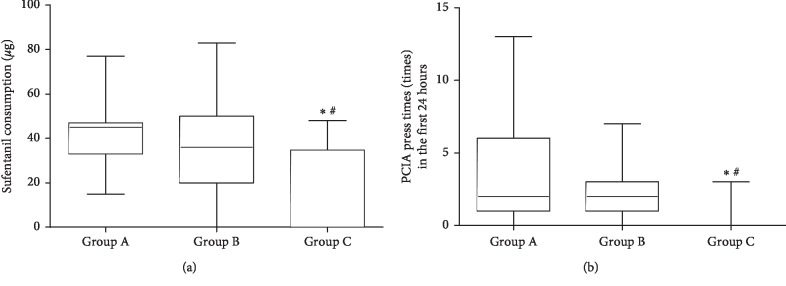
(a). The comparison of the sufentanil consumption (*μ*g) in the first 24 hours among the three groups (median (quartiles)). Significantly less sufentanil consumption in Group C than in Group A and Group B. ^*∗*^Comparison between Group C and Group A, *p* < 0.05. ^#^Comparison between Group C and Group B, *p* < 0.05. (b). The comparison of PCIA press times (times) in the first 24 hours among the three groups (median (quartiles)). Significantly less PCIA press times in Group C than in Group A and Group B. ^*∗*^Comparison between Group C and Group A, *p* < 0.05. ^#^Comparison between Group C and Group B, *p* < 0.05.

In the first 24 hours postoperatively, the median cumulative sufentanil consumption in Group C was 0 (0–34.75) *μ*g, which was less than that in Group A (45.00 (33.00–47.00) *μ*g, *p*=0.005) and Group B (36 (20.00–50.00) *μ*g, *p*=0.023). The sufentanil consumption was similar between Group A and Group B (*p* > 0.05) (Figure 2(a)).

The results of the PCIA press times in the first 24 hours postoperatively are listed in Figure 2(b). Patients in Group C pressed the PCIA pump (0 (0–0) times) less times than patients in Group A (2 (1–6) times, *p* < 0.001) and Group B (2 (1–3) times, *p*=0.009). However, the comparison between Group A and Group B showed no significance (*p* > 0.05).

**Figure 3:**
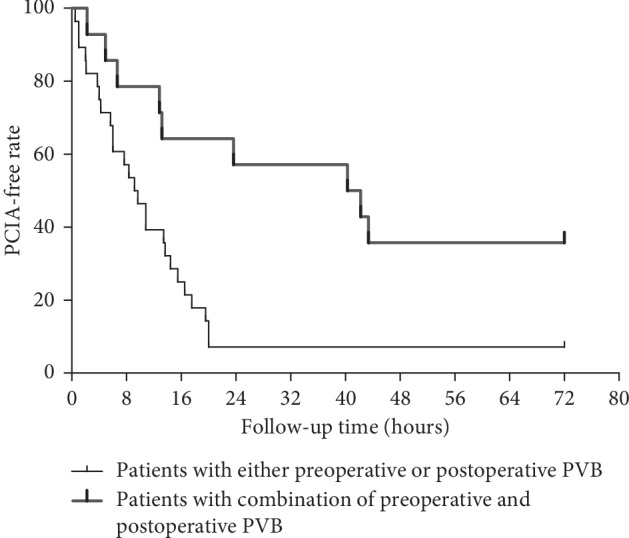
Kaplan–Meier survival analysis showed PCIA-free survival curve is different between patients with combination of preoperative and postoperative PVB (Group C) and patients with either preoperative or postoperative PVB (Group A + B), *p*=0.003.

Using Kaplan–Meier survival analysis, considering PCIA start as event, the PCIA-free rate of patients with combination of preoperative and postoperative PVB was 78.57%, 57.14%, 35.71%, and 35.71% for 8 h, 24 h, 48 h, and 72 h after surgery, respectively. In contrary, that of patients with either preoperative or postoperative PVB was 53.33% for 8 h after surgery and maintained at 6.67% for 24 h, 48 h, and 72 h after surgery. Patients with the combination of PVB had a better event-free survival curve than those received either technique alone as analyzed by the log-rank test (*p*=0.003, Figure 3).

The results of the VAS recorded in the early postoperative period are presented in Table 2. No matter during rest, during rotation of the homolateral arm, or while coughing, there were no significant differences among the three groups (*p* > 0.05).

Table 3 shows the rescue analgesia and complications during the PVB procedure and postoperatively. In Group A, there were 2 patients that needed intravenous flurbiprofen for rescue analgesia, and 2 patients suffered PONV. In Group B, 3 patients had hypotension postoperatively, and PONV occurred in 3 patients. Pleural puncture during PVB procedure was observed in 1 patient of Group B.

## 4. Discussion

The current study was performed to compare the analgesic efficacy of preoperative or postoperative PVB (Group A or Group B) versus the combination of both (Group C) in patients undergoing VATS. The results showed that patients in Group C had considerably less cumulative sufentanil consumption and PCIA press times in the first 24 hours postoperatively. Patients with combination of preoperative and postoperative PVB had a higher PCIA-free rate than patients with either technique alone.

Previous studies showed that preoperative or postoperative PVB can provide good analgesia [15] by blocking unilateral multisegmental spinal and sympathetic nerves [16]. Vogt found preoperative PVB seemed to prolong analgesic effect [12], which may be attributed to the fact that PVB before incision may provide a pre-emptive analgesic effect by reducing the nociceptive input to the central nervous system [17]. Moreover, the relatively sparse vascularity of the paravertebral space may slow the removal of local anesthetics, thus prolonging the duration of action [18]. However, the effectiveness in pain relief for patients undergoing VATS was similar between preoperative PVB and postoperative PVB [19]. Also, they did not compare the pain relief between the combination of preoperative and postoperative PVB and PVB performed either before or after surgery.

In the current study, patients receiving a combination of preoperative and postoperative PVB had significantly lower sufentanil consumption and less press times of PCIA in the first 24 postoperative hours. Furthermore, the Kaplan–Meier survival analysis of patients with the combination of preoperative and postoperative PVB had a significantly better PCIA-free survival curve, which indicated a delayed first sufentanil request time and a better analgesic effect. Except pre-emptive analgesic effect of preoperative PVB [17], this effect may also be ascribed to the late analgesia provided by postoperative PVB, the action time of which may be related to the pharmacokinetics of local anesthetics [20]. Besides, combination of preoperative and postoperative PVBs may improve the successful rate of block [15] and enhance the blocking effect for injecting twice the dose of ropivacaine [21].

The purpose of administering sufentanil in this study, one part of multimodal analgesia, was to reduce visceral pain mediated by the phrenic and vagus nerves [22]. Sufentanil infused by PCIA might mask the difference in regional analgesic efficacy among the three groups, resulting in no significant differences in VAS at any time point.

PVB can also be performed using a catheter technique. However, Chester C found there was no difference in the analgesia effectiveness between the single-shot PVB and continuous PVB [23]. Besides, some authors believed that continuous PVB using the landmark technique was not satisfactorily predictable and effective [24]. Even with the help of visualization technique, the possibility of locating the catheters in wrong positions could still be up to forty percent [25]. Also, a single-shot PVB might reduce the severity of pain for 48 h after VATS [12], which coincides with the period of severe pain sensations postoperatively [26]. Therefore, in order to ensure the analgesia effect, single-shot PVB rather than continuous PVB was chosen in our study.

The incidence of adverse events was low in all groups, and only one patient in Group B was observed to have pleural puncture. During the ultrasound-guided PVB procedure, visualization of the needle and the pleura may decrease the risk of pleura perforation [27]. Moreover, ultrasound guidance may confirm the local anesthetic spread in the paravertebral space by the confirmation of the entry of the needle tip into the paravertebral space and observing displacement of the pleura [27].

There were some limitations in the study. Firstly, the present study had a relatively small sample size, which may be underpowered to detect any differences in side effects or complications of postoperative analgesia among the three groups. Although main outcomes of the present study, sufentanil consumption and PCIA press times, have shown differences among the groups, further large sample studies in multiple centers are still warranted. Secondly, the aim of this study was to investigate the effect of a combination of preoperative and postoperative on the acute pain relieve of patients with VATS. So, we did not perform a long-term evaluation of postoperative chronic pain. Also, more data could be gathered to analyze the prognosis of patients.

## 5. Conclusions

In conclusion, the combination of both preoperative and postoperative PVB would provide better postoperative analgesia than either technique alone after VATS. Further work is required to investigate whether the combination of preoperative and postoperative PVB would have effects on postoperative chronic pain and other long-term outcomes.

## Figures and Tables

**Table 1:** Demographic and surgical data.

	Group A	Group B	Group C	*p* value
Number of patients	15	15	14	
Gender				0.951
Male	8 (53.33%)	7 (46.67%)	7 (50.00%)	
Female	7 (46.67%)	8 (53.33%)	7 (50.00%)	
Age (y)	53.00 (40.00, 58.00)	56.00 (51.00, 64.00)	55.50 (45.25, 66.25)	0.357
BMI (kg/m^2^)	22.40 ± 3.68	23.38 ± 3.79	23.56 ± 3.56	0.656
ASA I	2 (13.33%)	2 (13.33%)	2 (14.29%)	1
ASA II	13 (86.67%)	12 (80.00%)	10 (71.42%)	0.587
ASA III	0 (0%)	1 (6.67%)	2 (14.29%)	0.302
Operation time (min)	90.00 (70.00, 110.00)	85.00 (65.00, 145.00)	81.50 (79.50, 111.25)	0.921
Type of surgery				
Wedge resection	7 (46.67%)	10 (66.67%)	6 (42.86%)	0.420
Lobectomy	3 (20.00%)	4 (26.67%)	4 (28.57%)	0.912
Segmentectomy	4 (26.67%)	0 (0%)	4 (28.57%)	0.087
Others	1 (6.66%)	1 (6.66%)	0 (0%)	1
Number of ports				0.566
2	4 (26.67%)	5 (33.33%)	2 (14.29%)	
3	11 (73.33%)	10 (66.67%)	12 (85.71%)	
Duration of chest drain (days)	4 (3, 5)	4 (3, 6)	3.5 (3, 4.5)	0.426

There were no significant differences in demographic and surgical data among the three groups. Numerical data were presented as numbers (percentages). Variables with a normal distribution were presented as mean ± standard deviation (SD). Variables with a skewed distribution were presented as median (quartiles). BMI: body mass index, ASA: anesthesiologists' physical class.

**Table 2:** Comparison of VAS in the 72 h postoperative period among the three groups.

		Group A	Group B	Group C	*p* value
During rest	Before surgery	0 (0–0)	0 (0–0)	0 (0–0)	0.762
	1 h after surgery	0 (0–0)	0 (0–0)	0 (0–0)	0.165
	8 h after surgery	0 (0–2.25)	0 (0–2.00)	0 (0–1.25)	0.991
	24 h after surgery	2.00 (0–2.00)	2.00 (0–2.00)	2.00 (0–2.00)	0.981
	48 h after surgery	0 (0–2.00)	0 (0–2.00)	0.5 (0–1.25)	0.711
	72 h after surgery	0 (0–0)	0 (0–0)	0 (0–0.25)	0.560

During rotation of the homolateral arm	Before surgery	0 (0–0)	0 (0–0)	0 (0–0)	0.762
	1 h after surgery	0 (0–2.00)	0 (0–0)	0 (0–0)	0.140
	8 h after surgery	0 (0–2.50)	0 (0–2.00)	1.5 (0–2.00)	0.937
	24 h after surgery	2.00 (2.00–3.00)	2.00 (2.00–4.00)	2.00 (2.00–3.25)	0.877
	48 h after surgery	2.00 (0–2.00)	2.00 (2.00–2.00)	2.00 (1.00–2.00)	0.954
	72 h after surgery	0 (0–1.00)	0 (0–2.00)	0 (0–2.00)	0.689

While coughing	Before surgery	0 (0–0)	0 (0–0)	0 (0–0)	0.838
	1 h after surgery	0 (0–2.00)	0 (0–0)	0 (0–0.25)	0.071
	8 h after surgery	1 (0–2.50)	2 (2.00–4.00)	1.50 (0–2.25)	0.331
	24 h after surgery	2.00 (2.00–4.00)	4.00 (2.00–4.00)	2.00 (2.00–3.00)	0.053
	48 h after surgery	2.00 (2.00–4.00)	2.00 (2.00–4.00)	2.00 (2.00–2.25)	0.296
	72 h after surgery	2.00 (0–2.00)	2.00 (0–2.00)	1.50 (0–2.00)	0.331

The VAS (median (quartiles)) during rest, during rotation of the homolateral arm, and while coughing had no significant difference among the three groups in the 72 h postoperative period, *p* > 0.05.

**Table 3:** Rescue analgesia and complications during the PVB procedure and postoperatively.

	Group A	Group B	Group C
Rescue analgesia	2	0	0
Hypotension	0	3	0
Arrhythmia	0	0	0
PONV	2	3	0
Pruritus	0	0	0
Dizziness	0	0	0
Pleural puncture	0	1	0
Pneumonia	0	0	0

Data are presented as number of patients. PONV, postoperative nausea and vomiting.

## Data Availability

The data underlying the findings of the study could be obtained if a request is sent to the corresponding author's email: chengbaoli1979@zju.edu.cn.
